# High CD45 expression of CD8+ and CD4+ T cells correlates with the size of HIV-1 reservoir in blood

**DOI:** 10.1038/s41598-020-77433-z

**Published:** 2020-11-24

**Authors:** Stefan Petkov, Yonas Bekele, Tadepally Lakshmikanth, Bo Hejdeman, Maurizio Zazzi, Petter Brodin, Francesca Chiodi

**Affiliations:** 1 Department of Microbiology, Tumor and Cell Biology, Biomedicum, Karolinska Institutet, Solna vägen 9, 17165 Solna, Stockholm Sweden; 2 Division of Clinical Pediatrics, Department of Women’s and Children’s Health, Science for Life Laboratory, Karolinska Institutet, Stockholm, Sweden; 3 Department of Clinical Science and Education, Södersjukhuset, Unit of Infectious Diseases, Venhälsan, Södersjukhuset, Karolinska Institutet, Stockholm, Sweden; 4 Department of Microbiology and Virology, Policlinico S. Maria Alle Scotte, Siena, Italy; 5 Unit of Pediatric Rheumatology, Karolinska University Hospital, Stockholm, Sweden

**Keywords:** Immunology, Microbiology, Medical research

## Abstract

Using mass cytometry, we investigated the expression of 28 markers on CD8+ and CD4+ T cells from HIV-1 infected patients with a variable size of HIV-1 reservoir defined as high (HR) and low (LR) reservoir; we aimed at identifying phenotypic associations of T cells with size of HIV-1 reservoir. We showed that the frequency of CD45+ CD8+ and CD4+ T cells was directly proportional to the size of HIV-1 reservoir; HR patients had a significantly larger frequency of blood CD45^high^ T cells and higher CD45 expression on both CD8+ and CD4+ T cells. CD45 is a receptor-type protein tyrosine phosphatase essential in TCR signaling. Functional and phenotypical analysis of CD45^high^ cells revealed that they express activation and proliferation markers (CD38 + HLA-DR + and Ki-67) and produce cytokines upon in vitro activation. CD45^high^ T cells also expressed high levels of immune check-point PD-1. Our results link CD45 expression on T cells to HIV-1 reservoir; PD-1 expression on CD45^high^ T cells may contribute to their exhaustion.

## Introduction

Current antiretroviral therapy (ART) regimens are highly effective in suppressing plasma HIV-1 RNA levels under the detection limits for the duration of their administration. However, in view of its ability to integrate into the genomes of host cells, HIV-1 forms long-lasting cellular reservoirs in which the virus is present in a latent form^1^. The viral reservoir was previously described to be comprised by CD4 + central memory T cells, where the virus is maintained due to the longevity of these cells and slow proliferation as a result of low antigen stimulation. In treated patients pro-viral sequences were also detected in CD4 + transitional memory T cells^2^. Single-cell characterization combined with quantification of translation-competent viral reservoirs recently revealed a broad phenotypical diversity of cells harbouring HIV-1 reservoirs including T cells with effector functions^3^. Additional cell types, such as macrophages and astrocytes, can contribute to the viral reservoir^4^.

There are multiple theories outlining the establishment of HIV-1 latency^5–8^ and there is an agreement that the viral reservoir is seeded very early during infection^9^. Initiation of ART as early as 10 days post primary infection does not prevent establishment of latency in CD4 + T cells despite the achieved control of viremia^10^. Even though the formation of the HIV-1 reservoir cannot be prevented by ART, there are numerous reports showing that its early initiation is associated with a reduced size of the reservoir^11,12^. Moreover, an accelerated decay of the reservoir has been observed in patients who started ART early during acute infection^13^. Despite an intense search by the HIV-1 community for biomarkers characterizing HIV-1 reservoirs, the phenotype of reservoirs remains elusive. CD32a, the Fc gamma receptor IIa, has been proposed to represent a marker for HIV-1 reservoirs due to its association with a prominent enrichment in HIV-1 DNA^14^; this finding has however been challenged by several groups and the different results can be explained in the difficulty of obtaining substantial numbers of CD32a + CD4 + T cells to verify the connection of this marker with the HIV-1 reservoir.

CD8 + T cells play a critical role in control of HIV-1 infection. Following acute HIV-1 infection, a drop in viremia occurs only after the emergence of virus-specific CD8 + T cells^15^. Furthermore, natural control of infection without ART intervention in elite controllers is attributed to features of CD8 + T cells including expression of certain MHC-I alleles^16^. During ART administration, the latent reservoir is not accessible to immune surveillance since most cells harbouring latent provirus are quiescent and do not express viral antigens. It has been shown in many contexts (and reviewed in^17^) that ART is associated with decreased HIV-1 specific CD8 + T cells responses, in patients initiating therapy in both acute and chronic infection. However, a proportion of latently infected cells do transiently express viral antigens during proliferation or activation, which makes them targets for a cytotoxic T cell response^18^, emphasizing the importance of CD8 + T cells as means for controlling the size of the viral reservoir.

HIV-1 induced immune dysfunctions are only partially reversed by ART. Complete suppression of circulating viremia and partial restoration of CD4 + T cell counts is achieved in patients under therapy. Abnormalities of CD4 + and CD8 + T cells have however been reported even in patients who initiated ART in the acute phase of the infection^12,19^. CD4 + T cell loss in the mucosa of the gastrointestinal tract, a critical compartment for the development of HIV-1 immunopathology, occurs regardless of the timing of ART introduction^20^. CD8 + T cells of patients treated within a few weeks from HIV-1 acquisition or in the chronic phase of infection both exhibit phenotypic abnormalities including upregulation of exhaustion markers^21^ and soluble markers of inflammation can still be detected in these patients^22^. The detailed mapping of these phenotypical aberrations in treated patients is crucial and can help the development of improved treatment protocols.

Flow cytometry has traditionally served as the primary method for performing phenotypic analyses of immune cells. Mass cytometry has recently gained a lot of attention because of its capacity of simultaneously capturing a wide array of markers^23^. In this study we used mass cytometry to assess the relationship of markers expressed on CD4+ and CD8+ T cells from HIV-1 infected patients with the size of virus reservoirs. We measured the size of the viral reservoir in HIV-1 infected patients who had initiated ART at either acute or chronic phases of infection^19^ and arbitrarily assigned them to two groups representing the lower (LR) and higher (HR) spectrum of the virus reservoir size. The high expression of the CD45 molecule on CD8 + and CD4 + T cells positively correlated with the size of virus reservoirs.

## Results

### Frequencies of CD8 + and CD4 + T cells in HR and LR patients

**Figure 1:**
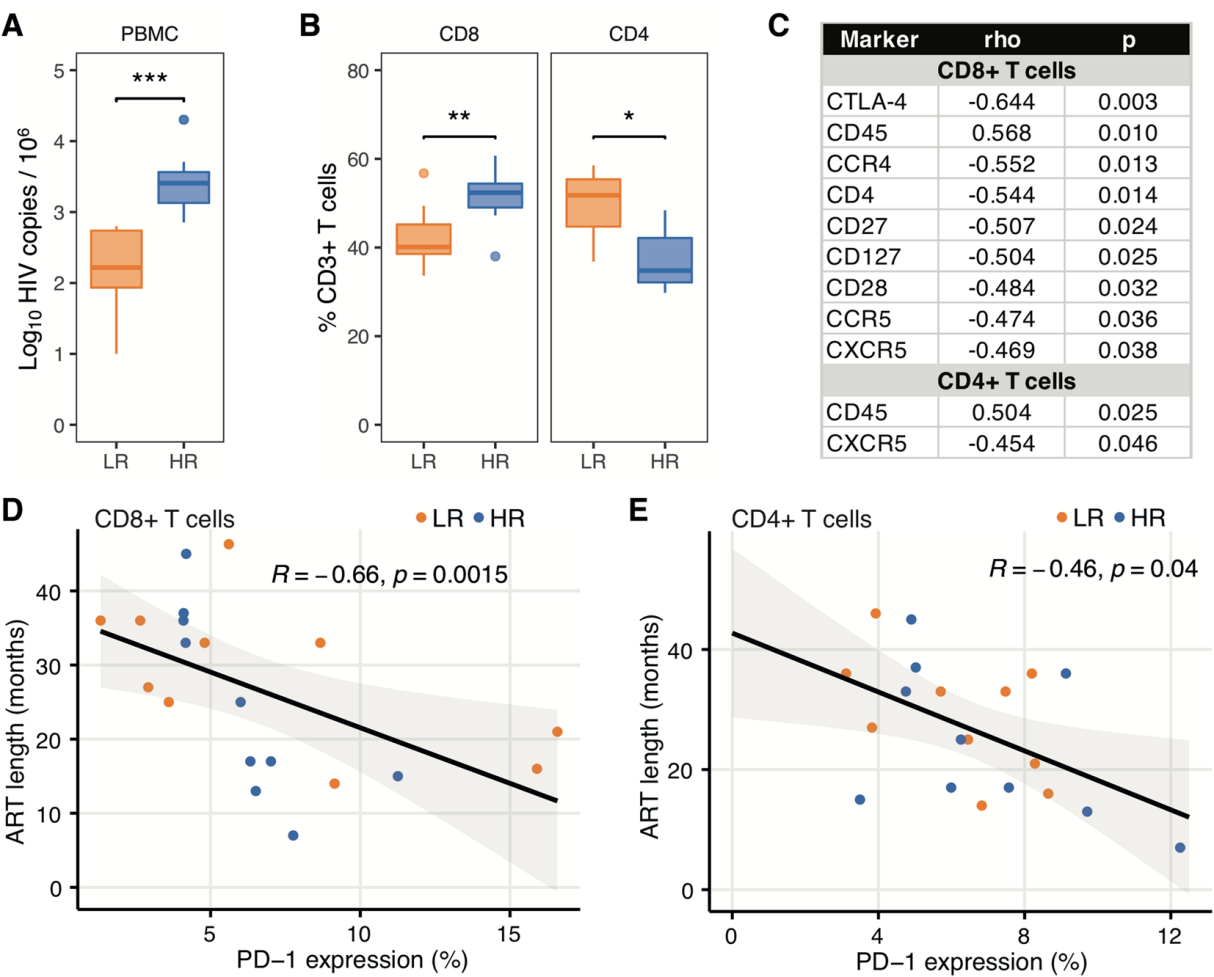
Number of HIV-1 DNA copies, frequencies of CD8 + and CD4 + T cells, and proteins correlating with the size of the HIV-1 reservoir and ART length in LR and HR patients. (**A**) Number of HIV-1 DNA copies in 10^6 ^PBMCs. (**B**) Frequencies of CD8 + and CD4 + T cells are shown. (**C**) Markers detected by CyTOF which correlate with the size of HIV-1 reservoir in the whole group of HIV-1 infected patients. (**D**,**E**) Correlation of ART length in LR and HR patients with frequencies of PD-1 + CD8 + and CD4 + T cells. Blood specimens from 10 HIV-1 infected patients with low HIV-1 reservoirs (LR) and 10 patients with high reservoirs (HR) were used. *p < 0.05, **p < 0.01, ***p < 0.001.

The patients were arbitrarily divided into two groups using the median point of the reservoir size in our cohort (673.5 HIV-1 DNA copies/10^6^ PBMCs) as a threshold between the low reservoir (LR; 10 patients) and high reservoir (HR; 10 patients) groups. The median value of the virus reservoir was 171 (range 10–631) in LR and 2570.5 (716–20.029) in HR patients (p < 0.001) (Fig. 1A). Clinical information on the patients is presented in Table 1. The median length of ART was not significantly different between the groups [median 30 (range 14–46) and 21 (7–45) months for LR and HR, respectively; p > 0.05]; the patients remained under virological control during ART administration. The frequencies of CD8 + T cells were significantly increased in HR patients compared to LR patients [median LR 40.1 (33.6–56.7); HR 52.4 (38.0–60.7), p < 0.01] (Fig. 1B) whereas the opposite trend was noticed for CD4 + T cells [median LR 51.7 (36.8–58.5); HR 34.8 (29.8–48.4), p < 0.05] (Fig. 1B).

**Table 1:** Clinical and immunological data of patients included in the study.

Group	Time of ART initiation	Number HIV-1 DNA copies	CD4 + T cell number	CD8 + T cell number	CD4 + /CD8 + ratio	Months on ART
LR	LA	< 10	890	1187	0.75	27
LR	EA	80	1340	957	1.40	14
LR	EA	86	600	556	1.08	21
LR	EA	89	580	382	1.52	36
LR	EA	126	1090	795	1.37	36
LR	EA	216	930	603	1.54	33
LR	EA	544	1280	955	1.34	16
LR	EA	548	1100	696	1.58	33
LR	LA	574	690	812	0.85	46
LR	EA	631	1110	941	1.18	25
HR	LA	716	840	1217	0.69	17
HR	LA	956	720	878	0.82	13
HR	EA	1341	760	559	1.36	7
HR	LA	1681	810	1173	0.69	36
HR	LA	2289	780	1083	0.72	25
HR	LA	2852	610	824	0.74	37
HR	LA	3135	960	980	0.98	15
HR	EA	3669	450	395	1.14	17
HR	LA	5102	980	1065	0.92	33
HR	LA	20.029	1000	990	1.01	45

*LR* low reservoir, *HR* high reservoir, *EA* early ART, *LA* late ART.

### The size of HIV-1 reservoir correlates with molecules expressed on CD4 + and CD8 + T cells

We performed mass cytometry (CyTOF) detecting 28 different markers (Supplementary Table 1) on PBMCs and several analyses were conducted to establish a phenotypic association of CD8 + T cells with the size of the virus reservoir. The correlation of CD8 + T cell frequency expressing each of the markers on the CyTOF panel with the size of the HIV-1 reservoir in the whole group of HIV-1 infected patients was analysed. This revealed that 9 markers had a statistically significant correlation with the size of the reservoir (Fig. 1C). The frequency of CTLA-4, CCR4, CD4, CD27, CD127, CD28, CCR5 and CXCR5 expressing CD8 + T cells inversely correlated with the number of HIV-1 DNA copies in PBMCs; only the frequency of CD45 + CD8 + T cells was directly proportional to the size of the HIV-1 reservoir. All molecules showed a high association with HIV-1 reservoirs (Fig. 1C).

The expression of CD45 on CD4 + T cells also directly correlated to the size of the reservoirs (Fig. 1C); on the other hand, CXCR5 expression on CD4 + T cells negatively correlated to the number of HIV-1 DNA copies in PBMCs.

The 20 patients included in the study comprise 10 individuals who started ART during the acute phase of the infection (EA = early ART) and 10 who started ART during the chronic phase of infection (LA = late ART). In order to assess whether the significant correlations shown in Fig. 1C were impacted by the time of ART initiation we stratified the cohort into EA (median size of HIV-1 reservoir: 380 copies; range 80–3669) and LA patients (median 1985 copies; range 10–20.029) and analysed the intragroup association between the reservoir size and the expression of CyTOF markers on CD4 + and CD8 + T cells (Table 2). The results presented in Table 2 reveal that the largest number of significant correlations with the size of the virus reservoir was found for markers expressed on CD8 + T cells when the patients were analysed as a single group as already reported in Fig. 1C.

**Table 2:** Correlation of the virus reservoir with CyTOF markers´ expression on CD8 + and CD4 + T cells isolated from patients starting ART at the acute and chronic phase of infection.

Marker	CD8 + T cells						CD4 + T cells					
	EA		LA		All patients		EA		LA		All patients	
	rho	p	rho	p	rho	p	rho	p	rho	p	rho	p
CCR4	− 0.685	**0.035**	− 0.224	0.537	− 0.552	**0.013**	− 0.261	0.470	− 0.273	0.448	− 0.359	0.120
CCR5	0.018	0.973	− 0.467	0.178	− 0.474	**0.036**	− 0.055	0.892	− 0.333	0.349	− 0.430	0.060
CCR6	0.067	0.865	0.285	0.427	− 0.367	0.112	− 0.236	0.514	0.321	0.368	− 0.123	0.603
CD127	− 0.321	0.368	− 0.564	0.096	− 0.504	**0.025**	0.042	0.919	0.018	0.973	− 0.165	0.484
CD161	− 0.285	0.427	0.527	0.123	− 0.132	0.577	− 0.467	0.178	0.309	0.387	− 0.101	0.672
CD27	− 0.418	0.232	− 0.673	**0.039**	− 0.507	**0.024**	− 0.636	**0.054**	− 0.248	0.492	− 0.340	0.143
CD28	− 0.418	0.232	− 0.648	**0.049**	− 0.484	**0.032**	− 0.515	0.133	− 0.139	0.707	− 0.377	0.102
CD31	− 0.055	0.892	− 0.152	0.682	− 0.242	0.302	− 0.103	0.785	0.127	0.733	− 0.095	0.691
CD38	0.103	0.785	0.079	0.838	0.011	0.967	− 0.248	0.492	− 0.006	1.000	0.146	0.538
CD39	0.479	0.166	0.564	0.096	0.128	0.590	0.539	0.113	0.273	0.448	− 0.003	0.992
CD4	− 0.624	0.060	0.091	0.811	− 0.544	**0.014**	NA	NA	NA	NA	NA	NA
CD3	NA	NA	NA	NA	NA	NA	NA	NA	NA	NA	NA	NA
CD44	0.333	0.349	0.794	**0.010**	0.245	0.296	0.176	0.632	− 0.345	0.331	0.152	0.521
CD45	0.576	0.088	0.127	0.733	0.568	**0.010**	− 0.224	0.537	0.248	0.492	0.504	**0.025**
CD45RA	− 0.406	0.247	0.539	0.113	0.030	0.901	0.515	0.133	− 0.152	0.682	− 0.140	0.555
CD5	0.564	0.096	− 0.321	0.368	0.164	0.488	0.370	0.296	0.321	0.368	0.155	0.513
CD57	0.382	0.279	0.576	0.088	0.436	0.056	− 0.406	0.247	0.030	0.946	0.420	0.067
CD8	NA	NA	NA	NA	NA	NA	− 0.176	0.632	0.067	0.865	− 0.105	0.658
CTLA-4	− 0.745	**0.018**	− 0.297	0.407	− 0.644	**0.003**	− 0.600	0.073	− 0.091	0.811	− 0.368	0.111
CXCR3	0.406	0.247	0.455	0.191	0.275	0.239	0.503	0.143	0.333	0.349	0.395	0.085
CXCR5	− 0.176	0.632	− 0.067	0.865	− 0.469	**0.038**	− 0.527	0.123	− 0.067	0.865	− 0.454	**0.046**
HLA-DR	0.358	0.313	− 0.188	0.608	0.005	0.987	0.152	0.682	− 0.624	0.060	− 0.020	0.937
ICOS	− 0.248	0.492	0.103	0.785	− 0.233	0.321	0.018	0.973	− 0.152	0.682	− 0.095	0.691
Ki-67	− 0.006	1.000	0.152	0.682	0.250	0.287	− 0.539	0.113	0.164	0.657	− 0.268	0.253
PD-1	− 0.152	0.682	0.091	0.811	0.002	0.997	0.212	0.560	0.103	0.785	− 0.084	0.724
TCRab	− 0.442	0.204	0.236	0.514	− 0.101	0.672	− 0.030	0.946	− 0.042	0.919	− 0.030	0.901

Non T cell-specific markers, CD19 and CD11c, were excluded.

*EA* early ART at acute infection, *LA* late ART at chronic infection, *NA* not applicable.

We also evaluated whether a correlation existed between ART treatment length with the size of reservoirs, clinical and immunological parameters and markers included in CyTOF panel. Significant inverse correlations were found between length of ART treatment and the frequencies of PD-1 + CD8 + T cells (Fig. 1D) and PD-1 + CD4 + T cells (Fig. 1E), suggesting an impact of ART length in the decrease expression of checkpoint molecule PD-1 on T cells.

### Unique CD8 + and CD4 + T cell clusters distinguish LR and HR patients

**Figure 2:**
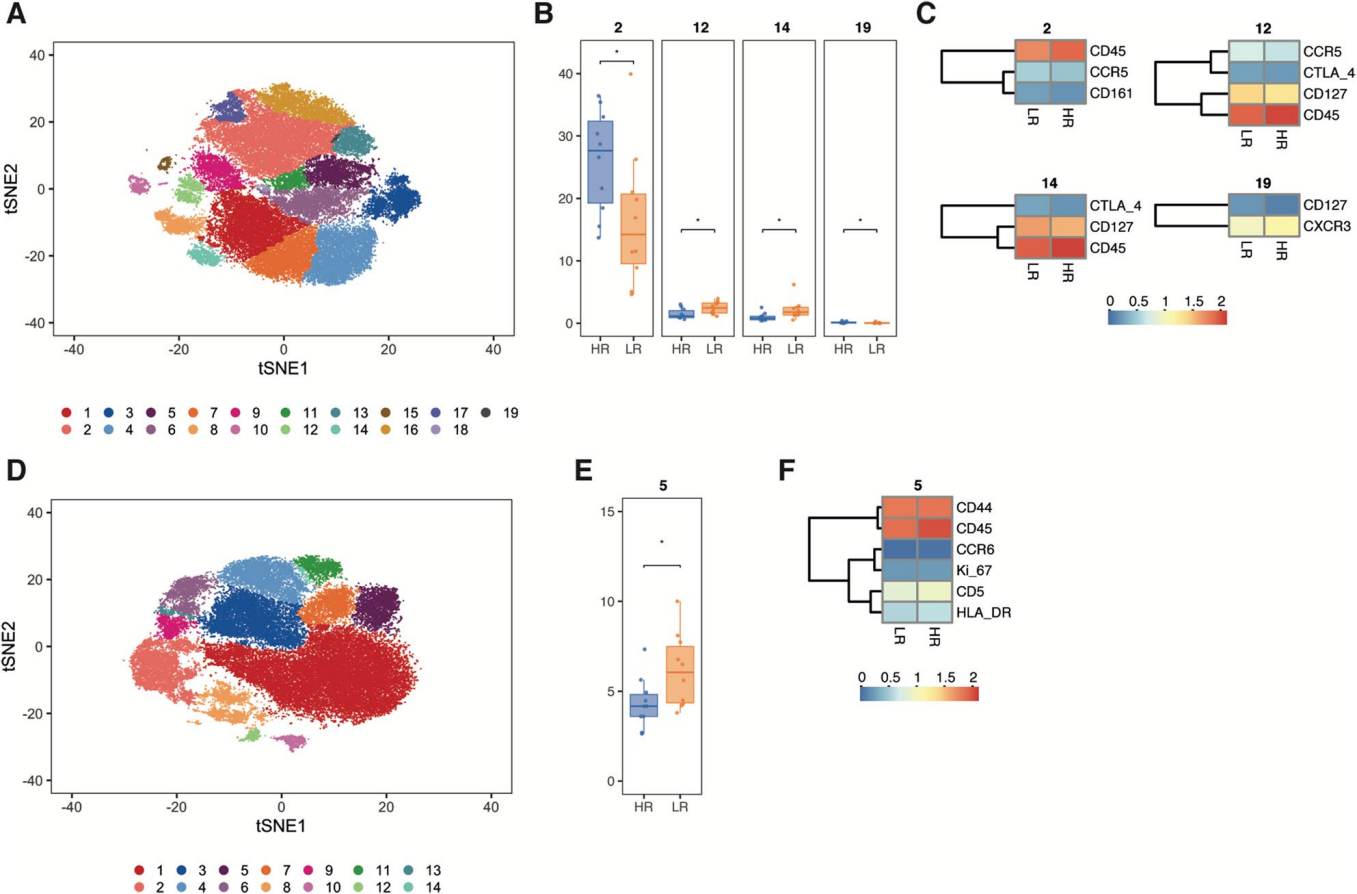
tSNE maps of gated CD8 + and CD4 + T cells, cluster abundance and marker expression within differentially regulated clusters. (**A**) Visualization of CD8 + T cell clustering on the tSNE space. (**B**) Comparison of cluster abundance within the CD8 + T cell populations of LR (n = 10) and HR (n = 10) patients. Statistically significant differences are indicated by asterisks. (**C**) Marker expression within differentially regulated clusters of CD8 + T cells. (**D**) Visualization of CD4 + T cell clustering on the tSNE space. (**E**) Comparison of cluster abundance within the CD4 + T cell populations of LR and HR patients. (**F**) Marker expression within differentially regulated clusters of CD4 + T cells. The heatmaps represent only clusters whose abundance was significantly different between the LR and HR groups. *p < 0.05.

We used t-stochastic network embedding (tSNE) to perform dimensionality reduction of CD8 + and CD4 + T cell populations. The tSNE maps visualize the distribution of T cells expressing different lineage, differentiation and activation markers. The resulting tSNE maps were clustered by an algorithm allowing the detection of non-spherical clusters based on the density of the data points in two-dimensional data as implemented by the clusterX package^24^. This method automatically identified 19 CD8 + T cell clusters (Fig. 2A), which were characterized by distinct marker expression profiles.

We compared the abundance of each CD8 + T cell cluster between the LR and HR groups and detected 4 clusters of different abundances (Fig. 2B). The cells comprising these clusters represented approximately 30% of the overall CD8 + T cell population in both LR and HR patients. The expression of CD45, a key factor in TCR signalling, was found to be higher in HR compared to LR samples in 3 out of 4 clusters (Fig. 2C) whereas the expression of CD127, interleukin-7 receptor-α subunit, was reduced in 3 of the 4 clusters.

The tSNE analyses of CD4 + T cells revealed 14 clusters (Fig. 2D) of which one was significantly more abundant in LR (Fig. 2E). This cluster comprised between 5–10% of the CD4 + T cells. Analysis of the markers expressed by the cluster revealed that CD45 expression was significantly higher for HR patients. Other markers that were slightly upregulated in the HR group in this cluster included CD44, CD5, CCR6, HLA-DR and Ki-67 (Fig. 2F).

To quantify the overall dissimilarity between CD8 + and CD4 + T cells in LR and HR patients we used the Jensen-Shannon divergence (JSD). Comparing the CD8 + T cell tSNE maps of LR and HR groups yielded a JSD score of 0.07, which confirmed differences in the distribution of CD8 + T cell phenotypes between LR and HR patients (Supplementary Fig. 1A). The JSD divergence test revealed some degree of diversity for CD4 + T cells (JSD = 0.04) (Supplementary Fig. 1B).

### High CD45 expression by CD8 + and CD4 + T cells is associated with increased size of the virus reservoir, cellular activation and exhaustion

**Figure 3:**
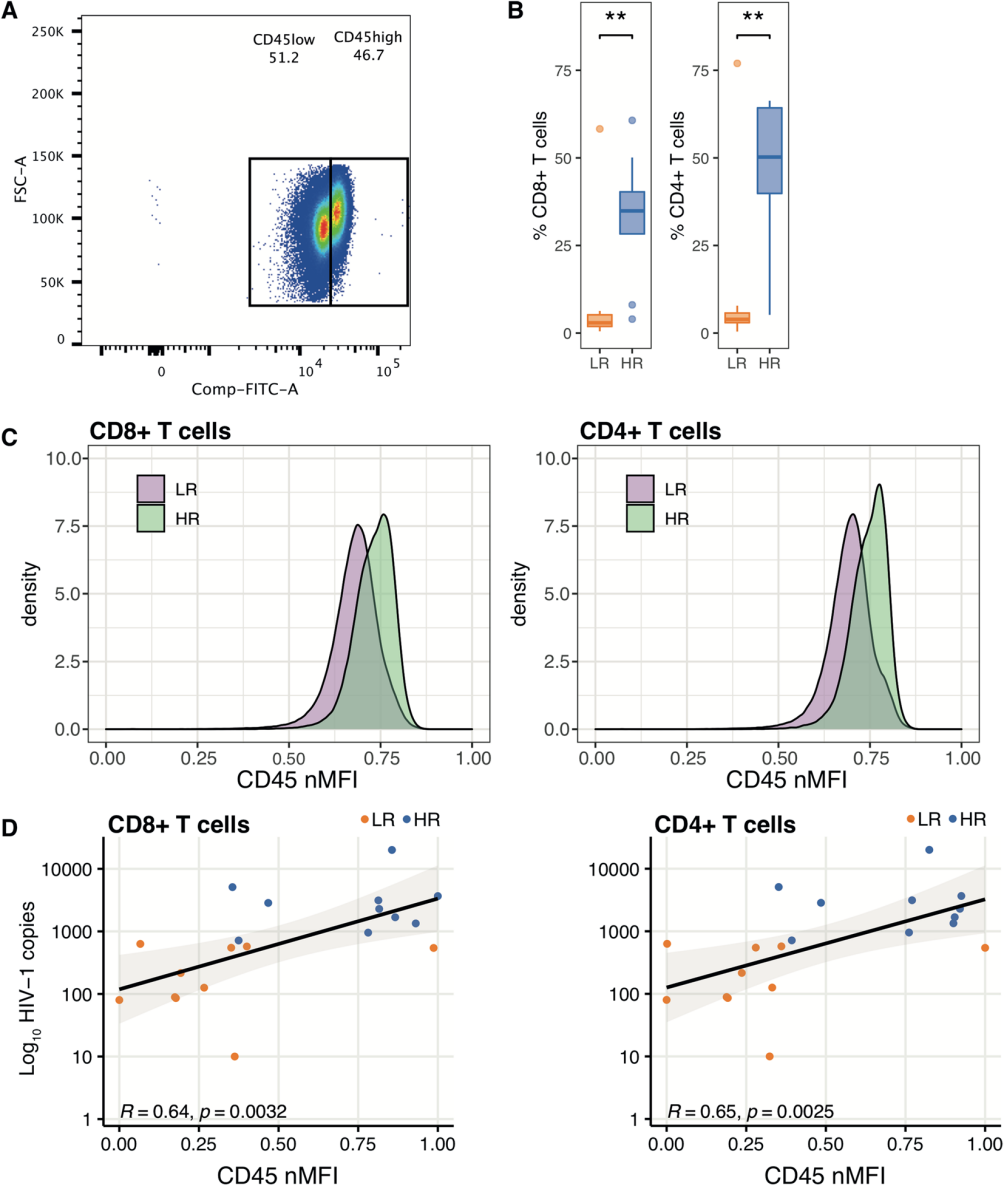
Expression of CD45 in CD4 + and CD8 + T cells and correlation of CD45 expressing T cells with the size of the virus reservoir. (**A**) Demonstration of the CD45^high^ and CD45^low^ T cell gating approach in flow cytometric data (shown for CD8 + T cells). (**B**) Frequency of CD45^high^ CD8 + and CD4 + T cells in patients with high (HR) and low reservoir (LR) size. (**C**) CD45 expression shown as normalized mean fluorescence intensity (nMFI) in CD8 + and CD4 + T cells of HR and LR patients. (**D**) Correlation between the size of the virus reservoir (DNA copies/10^6^ PBMCs) and CD45 expression (nMFI) in CD8 + and CD4 + T cells of HR and LR patients.

As high expression of CD45 was a characteristic of individual clusters distinguishing between LR and HR patients, we next attempted to correlate CD45 expression on CD8 + and CD4 + T cells with the virus reservoir; a demonstration of the approach used to gate T cells showing high and low CD45 expression is shown in Fig. 3A. We calculated the frequency of CD45^high^ CD4 + and CD8 + T cells and found that HR patients had a significantly larger frequency of both CD45^high^ CD4 + and CD8 + T cells in circulation (p < 0.01, Fig. 3B).

The CD45 mean fluorescence expression (MFI) was higher in CD8 + and CD4 + T cells of HR patients compared to that found in LR patients (Fig. 3C). In addition, the mean CD45 MFI of CD8 + and CD4 + T cells significantly correlated with the number of HIV-1 DNA copies in the whole group of patients (Fig. 3D).

**Figure 4:**
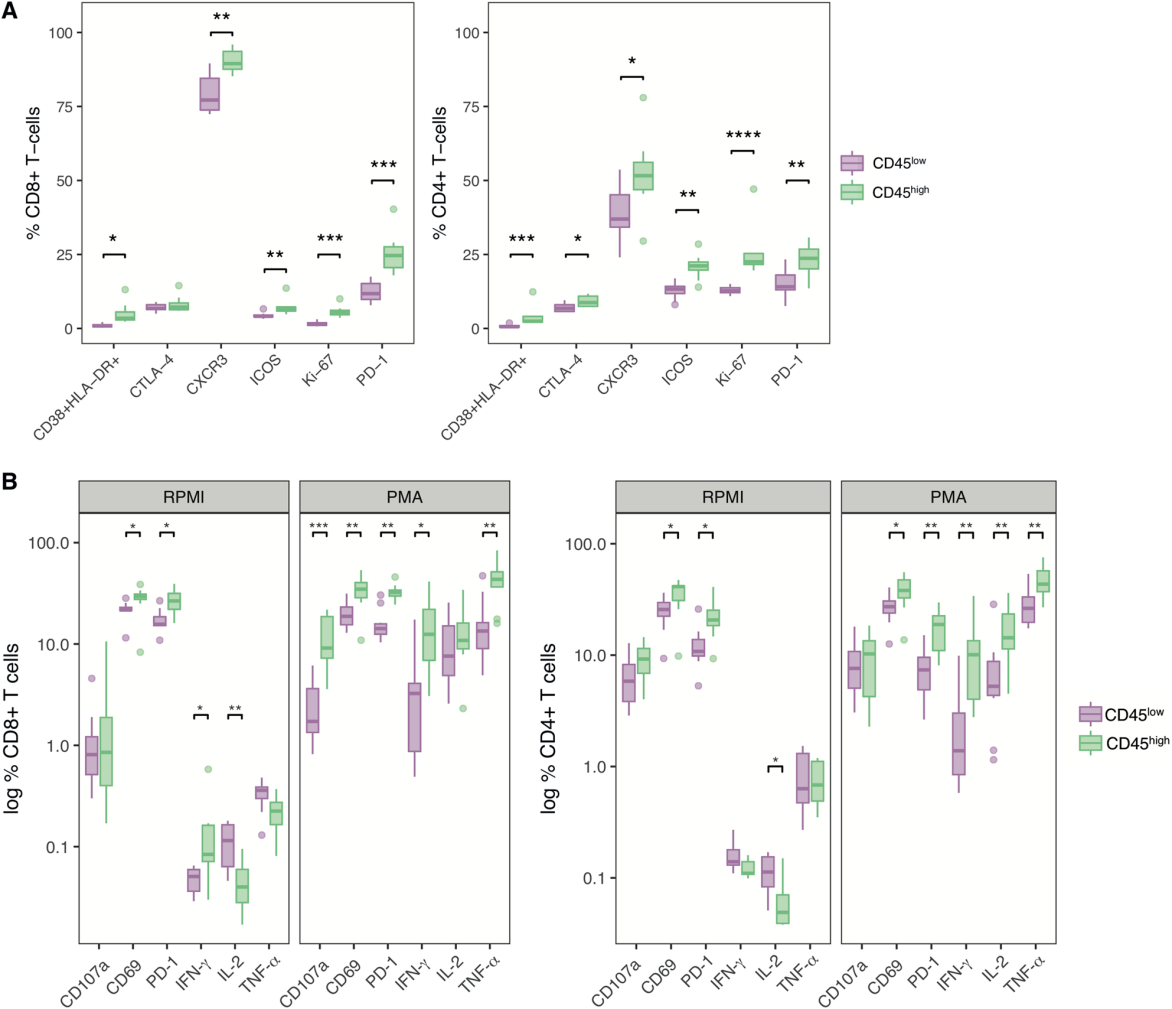
Phenotypical and functional characterization of CD45^low/high^ CD8 + and CD4 + T cells in LR and HR patients. (**A**) Comparison of the expression of CyTOF markers of T cell activation, proliferation and inhibition between CD8 + and CD4 + T cells with low and high CD45 expression. (**B**) Expression of activation markers and intracellular cytokines in CD8 + and CD4 + T cells from 8 HIV-1 infected patients were analysed by flow cytometry. The markers were assessed in steady-state conditions (RPMI) and after an overnight culture in the presence of PMA/Ionomycin (PMA). *p < 0.05, **p < 0.01, ***p < 0.001, ****p < 0.0001.

A critical question is whether the level of CD45 expression by CD8 + T cells of HIV-1 patients was associated to additional phenotypical markers. We examined the expression of markers of activation, proliferation and inhibition detected by CyTOF in CD45^low^ and CD45^high^ CD8 + T cells in HR patients. The frequency of CD38 + HLA-DR + cells, a classical indicator of abnormal activation during HIV-1 infection^25^, was significantly higher among CD45^high^ CD8 + T cells (Fig. 4A). Additionally, CXCR3, ICOS and Ki-67 molecules, which are known to be upregulated during activation^26–28^, were also more abundant in CD45^high^ T cells. The expression of the inhibitory signal CTLA-4 was not different, whereas PD-1 levels were higher in CD45^high^ CD8 + T cells. A similar pattern in the expression of these markers was observed for CD4 + T cells, which in addition showed an upregulation of CTLA-4 expression in the CD45^high^ group (Fig. 4A).

Finally, using intracellular cytokine staining we studied the functional capacity of CD45^low/high^ CD8 + and CD4 + T cells from 8 HIV-1 infected patients to produce cytokines in response to PMA/Ionomycin stimulation. To obtain a more complete understanding of the activation profile of these cells we also included markers of degranulation (CD107a) and activation (CD69). PD-1 was also included for consistency with prior experiments.

Upon PMA stimulation there was no change in the frequencies of CD45^high/low^ CD4 + and CD8 + T cells (results not shown). The base-line expression of PD-1, CD69 and IFN-γ was higher in CD45^high^ CD8 + T cells from HIV-1 infected patients (Fig. 4B); no differences in the expression of CD107a and TNF-α were observed. Stimulation with PMA/Ionomycin resulted in a marked increase in the expression of CD107a, IFN-γ, IL-2 and TNF-α by both CD45^high^ and CD45^low^ CD8 + T cells compared to their unstimulated controls; the levels of CD69 and PD-1 did not change significantly. Similar to the pattern observed at base-line, after stimulation the CD45^high^ cells expressed higher levels of cytokines IFN-γ and TNF-α, activation markers CD107a and CD69 and exhaustion marker PD-1.

A similar pattern of expression was detected for these markers in CD4 + T cells (Fig. 4B). CD69 and PD-1 were found to be upregulated at baseline in the CD45^high^ population compared to the CD45^low^ subsets; on the contrary, IL-2 expression was lower in CD45^high^ cells. Upon stimulation, the differences between CD45^high^ and CD45^low^ cells strengthened, with the exception of CD107a which was expressed at similar levels in both populations.

Overall, our observations suggest that a significant part of the T cell compartment in HR patients consists of CD45^high^ T cells that display high levels of activation and proliferation markers in presence of upregulated expression of the exhaustion marker PD-1.

## Discussion

The use of mass cytometry may offer a unique possibility to assess the relationship of markers present on T cells with the size of HIV-1 reservoirs; this approach does not target HIV-1 specific CD4 + and CD8 + T cells but may rather contribute to pin-point dysfunctional features of the whole T cell population during HIV-1 infection, which may indirectly affect the size of the reservoir. We asked whether any of the 28 molecules detected on CD8 + and CD4 + T cell populations showed a correlation with the number of HIV-1 DNA copies in PBMCs detected in low (LR) and high (HR) reservoir patients, analysed as a continuous variable. This approach revealed a strong indirect correlation between markers of differentiation (CD28, CD27 and CD127), immune modulation (CTLA-4) and homing (CCR4, CCR5 and CXCR5) on CD8 + T cells with HIV-1 DNA copies; the expression of CD45, on the other hand, was directly correlated to the size of the virus reservoir. In CD4 + T cells CD45 again directly correlated with HIV-1 reservoirs whereas CXCR5 expression was inversely correlated with this parameter.

We also addressed whether ART initiated during the chronic or acute phase of HIV-1 infection was associated to the markers present on CD8 + and CD4 + T cells in the CyTOF panel utilized in this work. These analyses revealed that the most significant correlations were detected when the virus reservoirs of all patients were analysed as one group in a continuous fashion. Interestingly the correlation of high CD45 expression on CD8 + T cells with the virus reservoirs only emerged when the patients were analysed in one group irrespectively of time of ART initiation.

A recently published work^29^ assessed the capacity of autologous CD8 + T cells from HIV-1 infected patients to eliminate HIV-1 reservoirs in CD4 + T cells treated with latency-reversing agents; the results of this work suggested that HIV-1 reservoirs harbouring replication-competent HIV-1 are characterized by an intrinsic resistance to elimination by CD8 + T cells. Our mass cytometry study identified some of the phenotypical characteristics of CD8 + T cells which may impair the capacity of CD8 + T cells to control the virus reservoir; interestingly all these molecules were previously identified in the context of functional properties of CD8 + T cells.

Gradual loss of CD28 on T cells has been shown to be the result of aging as well as prolonged antigenic stimulation during HIV-1 infection; effective ART administration has however been shown to shift this pathological parameter into a state where the majority of CD8 + T cells express CD28^30^. Down-regulation of CD127 occurs upon chronic immune activation via the TCR/CD28 pathway^31,32^ and has been previously observed in HIV-1 infection^33^. Reduced CD127 expression on CD8 + T cells from acutely infected patients correlated with the impaired capacity of CD8 + T cells to suppress HIV-1 replication^34^. CTLA-4 is a membrane protein with significant homology to CD28 which is primarily found on activated, but also regulatory, T cells; unlike CD28, CTLA-4 provides an inhibitory secondary signal resulting in suppression of T cell activation^35^.

In agreement with our results, the expression of CXCR5 on simian immunodeficiency virus (SIV)-specific CD8 + T cells has been previously shown to inversely correlate with viral load in chronically SIV infected rhesus macaques^36^. CCR5 plays an essential role in HIV-1 pathogenesis as the major coreceptor on CD4 + T cells used by HIV-1, yet the function of CCR5 on CD8 + T cells is not well understood. Interestingly, the expression of the chemokine receptor CCR5 correlates with functional CD8 + T cells in SIV-infected macaques^37^.

Among the molecules detected by mass cytometry on CD4 + T cells the expression of CXCR5 indirectly correlated to the size of HIV-1 reservoirs; CXCR5 expression in the periphery characterizes circulating T follicular helper cells^38^, which have been identified in multiple studies as HIV-1 reservoirs in untreated HIV-1 infection (reviewed in^39^); the dynamics of decay of HIV-1 reservoir in T_fh_ cells during ART are less studied. Also among CD4 + T cells CD45 expression correlated to the size of the reservoir.

CD45 is a receptor-type protein tyrosine phosphatase, which along with the tyrosine kinase Lck, plays an essential role in TCR signalling^40^. Binding of MHC-presented foreign antigens to TCR results in the phosphorylation of the TCR by the Lck kinase^41^, a process which can be restricted by high CD45 expression^42^. Hence the level of CD45 expression appears to be critical for the capacity of T cells to mount a specific immune response. Additionally, Cho et al. have recently emphasized the importance of CD45 in fine tuning the sensitivity of the TCR; by treating cells with CD45 inhibitors they were able to significantly increase the responsiveness to specific peptides^43^. Moreover, they also showed that CD45 expression is higher on memory CD8 + T cells, a subset which is crucial in mounting virus specific responses.

In our study, one major CD8 + T cell cluster, accounting for approximately 30% of the CD8 + T cell population, differed in its abundance between the LR and HR groups; in this cluster a significantly higher CD45 expression was found on CD8 + T cells of HR patients. The analysis of the CD4 + T cell cluster which was present at a significantly different abundance in HR and LR also showed that CD45 expression was higher on CD4 + T cells of HR patients.

From our results, it is evident that CD45 expression in CD4 + and CD8 + T cells is higher in patients with larger size of virus reservoir. This direct correlation of CD45 expression on CD8 + and CD4 + T cells with HIV-1 DNA copies is interesting and suggests that CD8 + T cells characterized by high CD45 expression, which may comprise HIV-1 specific CD8 + T cells, are unable to clear HIV-1 infected cells. Using the results obtained in mass cytometry we phenotypically characterized CD45^high^ and CD45^low^ CD8 + and CD4 + T cells from HR patients. CD45^high^ T cells, both CD4 + and CD8 + , presented with a strongly activated expression profile characterized by the high level of CD38 + HLA-DR+ ^44^ and other markers of immune activation such as Ki-67 and ICOS. We also observed a significantly higher level of PD-1 in CD45^high^ compared to CD45^low^ T cells. PD-1 is an important inhibitory receptor regulating exhaustion in CD8 + T cells in chronic infection and cancer^45,46^.

CD45^high^ T cells from HIV-1 infected patients, once stimulated in vitro, are fully capable to respond to activation: CD8 + by producing cytokines (IFN-γ, TNF-α) and upregulating markers of activation (CD107a, CD69); CD4 + by producing cytokines (IFN-γ, IL-2, TNF-α) and upregulating the expression of CD69. In both CD8 + and CD4 + CD45^high^ T cells a substantial up-regulation of PD-1 expression could be noticed reinforcing the association between high expression of CD45 and PD-1. High PD-1 levels can be rapidly induced following activation driven by TCR signalling. The inhibitory role of PD-1 expression on the function of CD8 + T cells was demonstrated by the administration of anti-PD-L1 antibody, which significantly reduced the time to clear lymphocytic choriomeningitis virus (LCMV) infection^47^. Similar results have been reported in herpes simplex virus infection, where blocking the PD-1 pathway enhanced effector T cell functions^48^. It has been recently suggested that PD-1 + exhausted T cells may display a complex phenotypical and functional patterns which may vary according to pathological conditions^49^. In the context of our study, PD-1 expression on CD8 + and CD4 + T cells indirectly correlated with the length of ART, likely reflecting a declined inflammation induced by treatment.

Recently Cavrois et al. used a 38-parameter mass cytometry phenotyping with dimensionality reduction and clustering to study how HIV-1 infection affects host gene expression and what CD4 + T cells support HIV-1 fusion and productive infection^50^. They were able to identify a subset of cells that support virus entry, but do not express any viral genes. These cells were long-lived lymphocytes characterized by high CD127 expression levels and could potentially constitute a long-term stable HIV-1 reservoir as IL-7 triggering through CD127 has been reported to drive the persistence of latently infected cells^2^.

The present study shows that high expression of CD45, previously implicated in inhibition of T cell activation^43^, on CD8 + and CD4 + T cells may be linked to the number of HIV-1 DNA copies present in HIV-1 reservoirs in circulation. The measurement of HIV-1 reservoir was conducted in our study by quantification of total HIV-1 DNA copies in PBMCs; a limitation of this analysis is that it detects both replication competent and defective HIV-1 proviruses. Recently published studies however indicated that the intact proviral DNA assay (IPDA), which detects replication competent HIV-1 provirus, correlates with levels of total and integrated DNA in blood and that total HIV-1 DNA copies also predict the outcome of the ex-vivo HIV-1 outgrowth assay which is consider the gold standard for estimating the HIV-1 reservoir size^51,52^. Functional studies with CD45 inhibitors in relevant in vitro and in vivo models of HIV-1 infection will provide information on whether a therapeutic reduction of CD45 expression on CD8 + T cells during HIV-1 infection may empower more efficient killing of HIV-1 infected cells by CD8 + T cells thus possibly leading to a reduced size of virus reservoirs.

## Methods

### Study participants

Twenty HIV-1 infected patients treated at the Department of Infectious Diseases, Södersjukhuset, Stockholm were enrolled. Patients were arbitrarily divided into two groups based on the median point of the reservoirs which was 673.5 HIV-1 DNA copies/10^6^ PBMCs and to ensure an even number of patients in both groups: the low reservoir (LR) group comprised 10 patients who had from 10 to 631 HIV-1 DNA copies/10^6^ PBMCs and the high reservoir (HR) group consisted of 10 patients with number of HIV-1 DNA copies spanning from 716 to 20.029. The patients were included in an earlier study assessing the effect of the time of ART initiation on immunopathology of CD4 + T cells^19^. 

At the time of sampling virus replication was suppressed in all patients. The RNA virus copies were under the limit of detection (< 20 copies/ml). The median age of patients was 42.5 for LR and 41.5 for HR patients. Each patient had been undergoing continuous ART for a median of 26 months, where the median duration of therapy was 30 months for LR and 21 for HR patients (p > 0.05). A list of relevant clinical parameters is shown in Table 1. Peripheral blood mononuclear cells (PBMCs) were isolated from 30 ml of patient blood and stored in 90% FBS/10% DMSO at − 196 °C until further analyses were performed.

### Measurement of total HIV-1 DNA copies in 10^6^ PBMC

PBMC DNA was obtained by manual extraction with the High Pure Viral Nucleic Acid Kit (Roche). Total PBMC HIV-1 DNA was quantified by using a homemade Taqman real time assay targeting a highly conserved region of the LTR gene and which methodological details were previously described^12^.

### Mass cytometry

Mass cytometry was performed as previously described^19^. PBMCs were thawed, resuspended in RPMI 1640 supplemented with 10% FBS, 1% penicillin and 25 U/ml benzonase and rested overnight in CO_2_ incubator at 37 °C. The cells were then stained for viability using 2.5 μM cisplatin (Fluidigm) for 5 min at room temperature (RT) after which the reaction was quenched with RPMI containing FBS. After resuspending in CyFACS (PBS with 0.1% BSA, 0.05% sodium azide, and 2 μM EDTA) buffer, 2 million cells of each sample were transferred into a 96-well round-bottom plate for staining. A 30 μl cocktail of surface antigen metal-conjugated antibodies (Supplementary Table 1) was added and the cell suspension was incubated at 4 °C for 30 min. Following two washes with CyFACS buffer, the cells were fixed overnight in 1% formaldehyde (FA) in PBS. The cells were then permeabilized using an intracellular fixation and permeabilization buffer set (eBiosciences) according to the manufacturer’s recommendations and then stained with 30 μl of intracellular antibody cocktail (Ki-67) for 60 min at RT. After the staining the cells were fixed in 1% FA at 4 °C.

Before acquisition, the samples were incubated for 20 min with 191/193Ir DNA intercalator (Fluidigm), which helps event acquisition by binding to nucleic acids. Finally, the cells were washed with PBS and Milli-Q water (MilliporeSigma) and then analyzed on a CyTOF 2 mass cytometer (Fluidigm) at a rate of 300–500 cells/second with noise reduction, a lower convolution threshold of 200, event length limits of 10–15 pushes, a sigma value of 3, and a flow rate of 0.045 ml/minute.

### Flow cytometry

Cryopreserved PBMCs were thawed and resuspended in RPMI 1640 supplemented with 10% FCS (R10), washed in PBS and then stained for 30 min at 4 °C with a mix containing anti-human monoclonal antibodies. The cells were then washed again and fixed in 4% PFA for 20 min at 4 °C. To perform the intracellular cytokine assay cells were rested for 4–5 h and then incubated overnight in the presence of R10 media supplemented with penicillin/streptomycin, GolgiPlug (#555029), GolgiStop (#554724) and an anti-CD107a monoclonal antibody. The leukocyte activation cocktail (#550583) containing PMA/Ionomycin was used for PBMCs stimulation. After overnight incubation, the cells were stained for surface markers, fixed/permeabilized using the Cytofix/Cytoperm kit (#554714, BD Biosciences) and then stained for intracellular cytokines. The following monoclonal antibodies were used to perform the flow cytometric assays: CD3/APC (#561811), CD8/PerCP-Cy5.5 (#565310), CCR7/PE-Cy7 (#557648), CD45/FITC (#555482), PD-1/CF-594 (#565024), CD107a (#561348), IFN-γ (#564039), IL-2 (#562914), TNF-α (#559321), CD69 (#555533) and a viability stain (cat. # L10119, Life Technologies, Carlsbad, CA). As isotype controls the following antibodies were used: IgG1/CF-594 (#562292), IgG1/PE-Cy7 (#557872), IgG1/BV711 (#56344), IgG1/BV421 (#562438), IgG1/PE (#554680), IgG1/APC (#555751). Reagents were purchased from BD Biosciences, unless stated otherwise. The samples were analysed using a BD LSR II flow cytometer.

### Data analysis

Flow cytometric data was analysed using FlowJo 10.4 (Becton Dickinson, Ashland, OR). CD45 high/low gating of the CD4 + /CD8 + T cell populations (Fig. 3) was performed using a biaxial plot with FITC-CD45 as the x-axis and forward scatter as the y-axis.

In the CyTOF experiments live cells were identified by the selection of DNA^hi^ and cisplatin^-^ events. The expression of all 28 markers (Supplementary Table 1) was analyzed in viable cells. The normalized and randomized and acrsinh-transformed data were imported in the R statistical environment for gating with *openCyto*^53^ using R Studio. High expression, as in CD45^high^, was defined by the distribution of events in the highest third of the range, which in this case was equal or higher than 2. CD3 + CD8 + and CD3 + CD4 + cells were gated and exported as FCS files for downstream analyses. The CD8 + and CD4 + T cell datasets were down sampled to 3000 cells from each donor. Dimensionality reduction was computed using *Rtsne*, the R implementation of the t-stochastic neighbour embedding algorithm^54^. The low-dimensional data were then clustered based on all the available markers using the *clusterX* package^24^. The produced tSNE maps were used to compare the differential abundance of clusters between the LR and HR groups. The *pheatmap* package was used to visualize the differential expression of cellular markers within the clusters^55^. Differences between the tSNE maps were also measured using the Jensen-Shannon divergence (JSD). We calculated the probability density function (PDF) with a kernel density estimation algorithm^56^ and then interpolated the discrete low-dimensional data. After the tSNE coordinates were converted to PDFs, the similarity between the maps was compared based on the corresponding PDFs. Base 2 logarithm was used in the computation of the JSD^57^ resulting in values ranging from 0, representing identical distributions, to 1 for disjoint distributions.

### Statistics

Comparing the cell frequencies, cluster abundance and MFI between the two groups was performed using a non-parametric unpaired Wilcoxon test. The correlation between the number of integrated virus copies and the frequencies of CD8 + and CD4 + T cell subpopulations were calculated using a spearman rank correlation test. Only p-values < 0.05 were considered as statistically significant.

### Study approval

The ethical committee at Karolinska Institutet (Stockholm, Sweden) reviewed and approved the study. All patients provided written informed consent before their participation in the study. All methods were performed in accordance with the relevant guidelines and regulations.

## Supplementary information

Supplementary Information
