# Supplementary material for: High CD45 expression of CD8+ and CD4+ T cells correlates with the size of HIV-1 reservoir in blood
Source: Sci Rep. 2020 Nov 24;10:20425. doi: 10.1038/s41598-020-77433-z (PMC7686502; doi:10.1038/s41598-020-77433-z)
Supplement: Supplementary file 1 — Supplementary Information [file 41598_2020_77433_MOESM1_ESM.pdf]

SUPPLEMENTARY INFORMATION

High CD45 expression of CD8+ and CD4+ T cells correlates with the size of HIV-1 reservoir in blood

Stefan Petkov[1], Yonas Bekele[1], Tadepally Lakshmikanth[2], Bo Hejdeman[3], Maurizio Zazzi[4], Petter Brodin[2,5], Francesca Chiodi[1]*

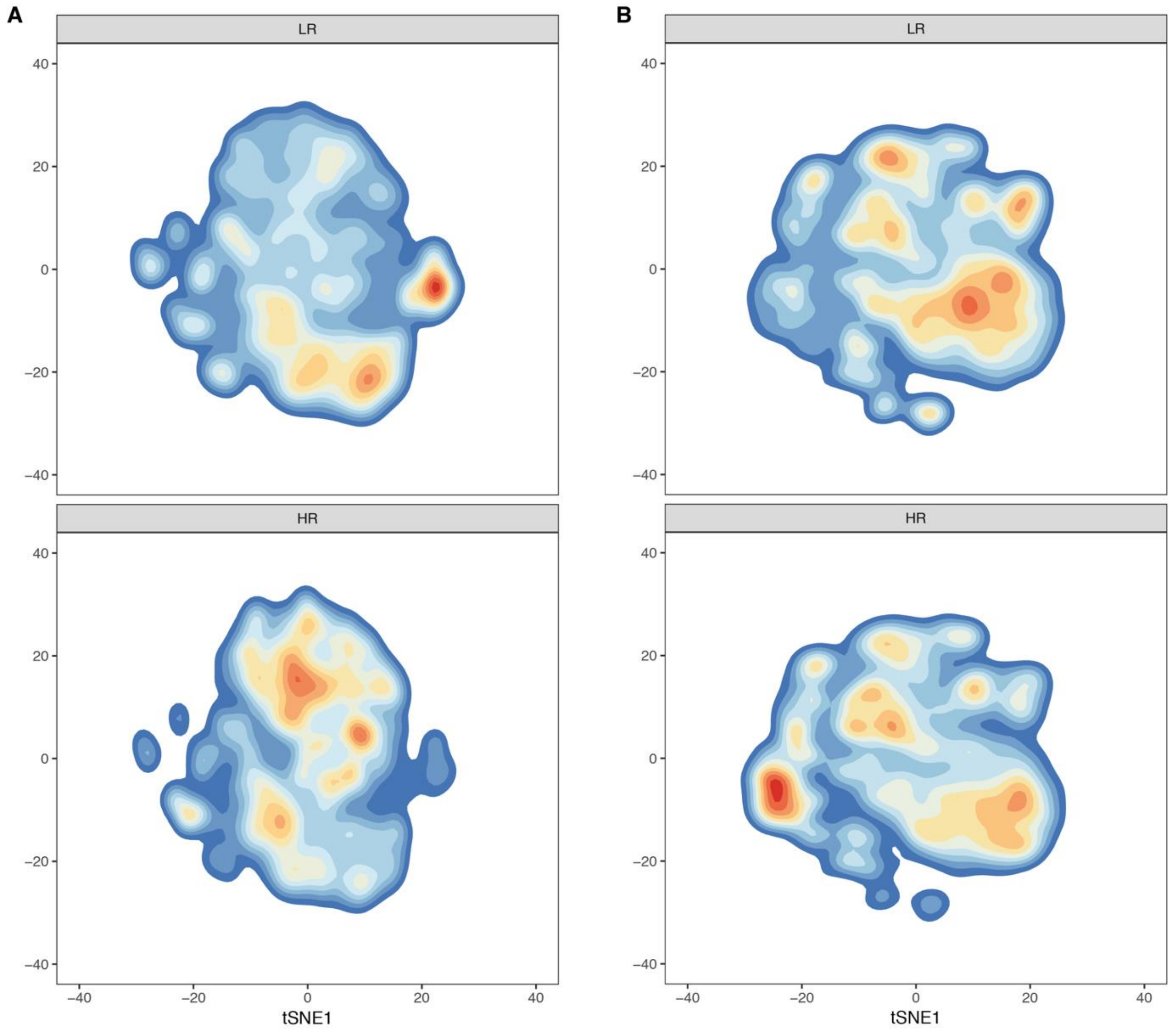

Supplementary Figure 1. Density plot overlay visualizing the distribution of events on the two-dimensional tSNE space in CD8+ and CD4+ T cells.

CD8+ T cells are shown in panel A and CD4+ T cells in panel B. The density plots display spatial differences between LR (up) and HR (down) groups. Blood specimens from 10 HIV-1 infected patients with low HIV-1 reservoirs (LR) and 10 patients with high reservoirs (HR) were used.

Supplementary Table 1. List of antibodies used in mass cytometry

#	Clone	Marker	Tag	Vendor
1	HI30	CD45	89Y	Fluidigm
2	HCD57	CD57	115ln	BioLegend
3	11A9	CCR6 (CD196)	141Pr	BD Biosciences
4	HIB19	CD19	142Nd	Fluidigm
5	UCHT2	CD5	143Nd	BioLegend
6	NP-6G4	CD195 (CCR5)	144Nd	Fluidigm
7	RPA-T4	CD4	145Nd	Fluidigm
8	SK1	CD8a	146Nd	BioLegend
9	Bu15	CD11c	147Sm	Fluidigm
10	WM59	CD31	148Nd	BioLegend
11	DX29	CD278 (ICOS)	151Eu	Fluidigm
12	IP26	TCRab	152Sm	BioLegend
13	UCHT1	CD3e	154Sm	Fluidigm
14	205410	CD194 (CCR4)	155Gd	R & D Systems
15	G025H7	CXCR3	157Gd	BioLegend
16	CD28.2	CD28	160Gd	BioLegend
17	HP-3G10	CD161	161Dy	BioLegend
18	B56	Ki-67	162Dy	Fluidigm
19	L243	HLA-DR	163Dy	BioLegend
20	BJ18	CD44	164Dy	BioLegend
21	A019D5	CD127	165Ho	Fluidigm
22	L128	CD27	167Er	Fluidigm
23	HIT2	CD38	168Er	BioLegend
24	HI100	CD45RA	169Tm	Fluidigm
25	14D3	CD152 (CTLA-4)	170Er	Fluidigm
26	EH12.2H7	CD279 (PD-1)	172Yb	BioLegend
27	A1	CD39	173Yb	BioLegend
28	51505	CXCR5	174Yb	R & D Systems
